# Human Salivary Micro-RNA in Patients with Parotid Salivary Gland Neoplasms

**DOI:** 10.1371/journal.pone.0142264

**Published:** 2015-11-06

**Authors:** Johannes H. Matse, Janice Yoshizawa, Xiaoyan Wang, David Elashoff, Jan G. M. Bolscher, Enno C. I. Veerman, C. René Leemans, Michiel D. Pegtel, David T. W. Wong, Elisabeth Bloemena

**Affiliations:** 1 Department of Oral and Maxillofacial Surgery and Oral Pathology, VU University medical center / Academic Centre for Dentistry Amsterdam (ACTA), Amsterdam, The Netherlands; 2 Department of Oral Biochemistry ACTA, University of Amsterdam and VU University Amsterdam, Amsterdam, The Netherlands; 3 School of Dentistry and Dental Research Institute, University of California Los Angeles, Los Angeles, California, United States of America; 4 Statistics Core, UCLA David Geffen School of Medicine, Division of General Internal Medicine and Health Services Research, University of California Los Angeles, Los Angeles, California, United States of America; 5 Department of Otolaryngology, VU University medical center, Amsterdam, The Netherlands; 6 Department of Pathology, VU University medical center, Amsterdam, The Netherlands; Kunming University of Science and Technology, CHINA

## Abstract

**Background:**

Currently, clinical examination, ultrasound scanning (with or without fine needle aspiration cytology), preoperative CT-scan and MRI are available for the differential diagnosis of parotid gland swelling. A preliminary non-invasive salivary diagnostic tool may be helpful in the clinical decision making process. Altered salivary micro-RNA (miRNA) expression levels have been observed in saliva from patients with various cancers. Therefore, we investigated miRNA expression levels in saliva samples from patients with a parotid gland neoplasm using Human miRNA cards in comparison to controls.

**Results:**

In the discovery phase, eight miRNAs were identified having different expression levels in patients compared to controls. In the validation phase, the differences in miRNA expression levels between patients and controls were confirmed for seven out of eight discovered miRNAs (p < 0.001). A combination of two miRNAs yielded a receiver-operator-characteristics curve with an AUC of 0.94 (95% CI: 0.87–1.00; sensitivity 91%; specificity 86%). Validation of discovered miRNAs in segregated collected parotid saliva revealed that expression of these miRNAs differ between whole saliva and parotid saliva.

**Conclusions:**

A two miRNA combination can predict the presence of a parotid gland neoplasm. Furthermore, this study suggested that the identified, patient-specific, salivary miRNAs were not derived from the parotid gland itself.

## Introduction

MicroRNAs (miRNAs) are small non-coding RNAs consisting of 19–25 nucleotides. They are involved in various cellular processes such as cell differentiation, proliferation and survival. MiRNAs inhibit translation by binding to complementary sequences in the 3’ UTR of multiple target mRNAs, usually resulting in their silencing [1,2]. Altered levels of miRNA expression have been implicated in the etiology of cancer [3,4].

Many studies have demonstrated the differential expression of miRNAs between cancer and the related normal surrounding tissue. Specific miRNA expression profiles were identified in a number of cancers [1,5–9]. Fluid-specific miRNA expression profiles have also been identified [1,10,11]. The expression of hsa-miR-21, hsa-miR-141 and the hsa-miR-200 family were up-regulated in serum from patients with ovarian cancer [12]. Furthermore, differences in miRNA expression profiles were found in cystic fluid of intraductal papillary mucinous neoplasm of the pancreas and in urine samples from patients with urinary bladder cancer. In saliva samples from patients with esophageal cancer, the expression level of three miRNAs (hsa-miR-10b*, hsa-miR-144, and hsa-miR-451) was significantly higher compared to the control group. Changes in miRNA expression levels have also been described in saliva samples from patients with oral squamous cell carcinoma [7,13–15]. The expression level of two miRNAs (hsa-miR-125a and hsa-miR-200a) was significantly lower in saliva from patients than in healthy control subjects [7]. Recently, we identified 6 potential biomarkers (mmu-miR140-5p, hsa-let-7g, hsa-miR-15b, hsa-miR-132, hsa-miR-222, and hsa-miR-374) that were differently expressed in whole saliva from patients with benign parotid neoplasms and malignant parotid neoplasms [16]. A combination of four miRNAs (hsa-miR-15b, hsa-miR-132, hsa-miR-223 and mmu-miR-140-5p) did discriminate between patients with a malignant tumor and those with a benign salivary gland tumor (sensitivity 69%, specificity 95%).

For clinical decision making, it is important to determine whether salivary gland swelling is due to a neoplasm or a reactive process. Nowadays, clinical examination and invasive methods such as ultrasound scanning (with or without fine needle aspiration cytology), preoperative CT-scan and MRI are the most common diagnostic methods used [17].

In the present study, we have investigated whether salivary miRNA expression profiles of whole saliva from patients with a parotid gland neoplasm differ from those of healthy controls. We found that the expression levels of seven validated miRNAs were higher in whole saliva from patients compared to controls. Using a combination of two of these, it was possible to discriminate between patients with a parotid gland neoplasm and controls. Furthermore, we found differences in the expression of the validated miRNAs between whole saliva and parotid saliva from the same patient.

## Materials and Methods

### Patients and salivary samples

#### Discovery phase

For the discovery phase, unstimulated whole saliva samples from 20 patients with a parotid gland neoplasm, obtained from the Salivary Gland Tumor Biorepository (SGTB) at the MD Anderson Cancer Clinic in Houston, TX, USA were tested. Data with regard to the miRNA expression in this group has already been described [16]. Ten unstimulated whole saliva samples from healthy controls were obtained from the Samsung Medical Center in Seoul, South-Korea (Table 1). No clinical information other than age, gender, ethnicity and tumor subtype was available.

**Table 1:** Patients’ characteristics. Patients’ characteristics of neoplasm samples and control samples used in the discovery and the validation phases.

Discovery phase in Whole saliva (n = 30)			Validation phase in Whole saliva (n = 60)			Validation phase in Parotid saliva (n = 26)		
	Neoplasm	Control		Neoplasm	Control		Neoplasm	Control
**Mean age (range)**	56.5 (33–82)	52.4 (39–67)	**Mean age (range)**	55.0 (20–89)	47.3 (23–70)	**Mean age (range)**	56.3 (55–90)	56.3 (55–90)
**sex (m/f)**	12/8	4/6	**sex (m/f)**	26/20	7/7	**sex (m/f)**	5/7	7/7
**Ethnicity**			**Ethnicity**			**Ethnicity**		
Hispanic	2	0	Hispanic	2	0	Hispanic	0	0
Caucasian	16	0	Caucasian	42	10	Caucasian	12	10
Black	2	0	Black	2	1	Black	0	1
Asian	0	10	Asian	0	3	Asian	0	3
**Neoplasm subtypes**			**Neoplasm subtypes**			**Neoplasm subtypes**		
Pleomorphic adenoma	6		Pleomorphic adenoma	19		Pleomorphic adenoma	7	
Warthins tumor	3		Warthins tumor	6		Warthins tumor	2	
Salivary duct carcinoma	2		Salivary duct carcinoma	6		Salivary duct carcinoma	3	
Muco-epidermoid carcinoma	1		Muco-epidermoid carcinoma	3				
Acinic cell carcinoma	1		Acinic cell carcinoma	3				
Adenoid cystic carcinoma	1		Adenoid cystic carcinoma	2				
Oncocytic carcinoma	1		Adeno-carcinoma, not otherwise specified	2				
Neuroendocine carcinoma	3		Basal cell adenocarcinoma	1				
Myoepithelial carcinoma	1		Basal cell adenoma	1				
Cystadeno-carcinoma	1		Myoepithelial carcinoma	1				
			Carcinoma ex-pleomorphic adenoma	1				
			Undifferntiate carcinoma	1				

#### Validation in whole saliva

The miRNAs emerging from the discovery phase were validated in an independent sample set of whole saliva. This set consisted of 32 randomly selected unstimulated whole saliva samples from patients with a parotid gland neoplasm and was obtained from the SGTB; 14 were collected at the Department of Otolaryngology and Head-Neck Surgery, VU University medical center, Amsterdam, The Netherlands.

Unstimulated whole saliva samples from 14 healthy controls were collected at the Department of Oral Biochemistry, Academic Centre for Dentistry Amsterdam, Amsterdam, The Netherlands (Table 1).

#### Validation in parotid saliva

Besides whole saliva, we also collected parotid saliva from 12 patients with a parotid gland neoplasm. Parotid saliva samples were collected separately from both the affected and the contralateral (normal) parotid gland using salivettes (Sarstedt, Nümbrecht, Germany) at the VU University medical center. As a control, parotid saliva was collected from 14 healthy controls. All parotid saliva was collected upon stimulation with 2% citric acid (Table 1).

### Salivary miRNA profiling

#### Discovery phase

MiRNA profiling of saliva samples from our previous study [16] was re-evaluated in comparison to the miRNA expression levels in saliva from healthy controls. In short, saliva samples were thawed and centrifuged. The cell-free supernatant (cleared saliva) was collected. Total RNA was isolated from cleared saliva using RNA extraction kits (Ambion mirVana Paris kit). Extracted RNA (1–10 ng) was reverse transcribed and pre-amplified. Undiluted pre-amplification product was mixed with 2x Taqman Master mix and loaded onto the Taqman Human MicroRNA cards (Applied Biosystems, Foster City CA). The cards with 750 miRNA were centrifuged and run on the Applied Biosystems 7900HT Fast Real-Time PCR instrument.

**Fig 1:**
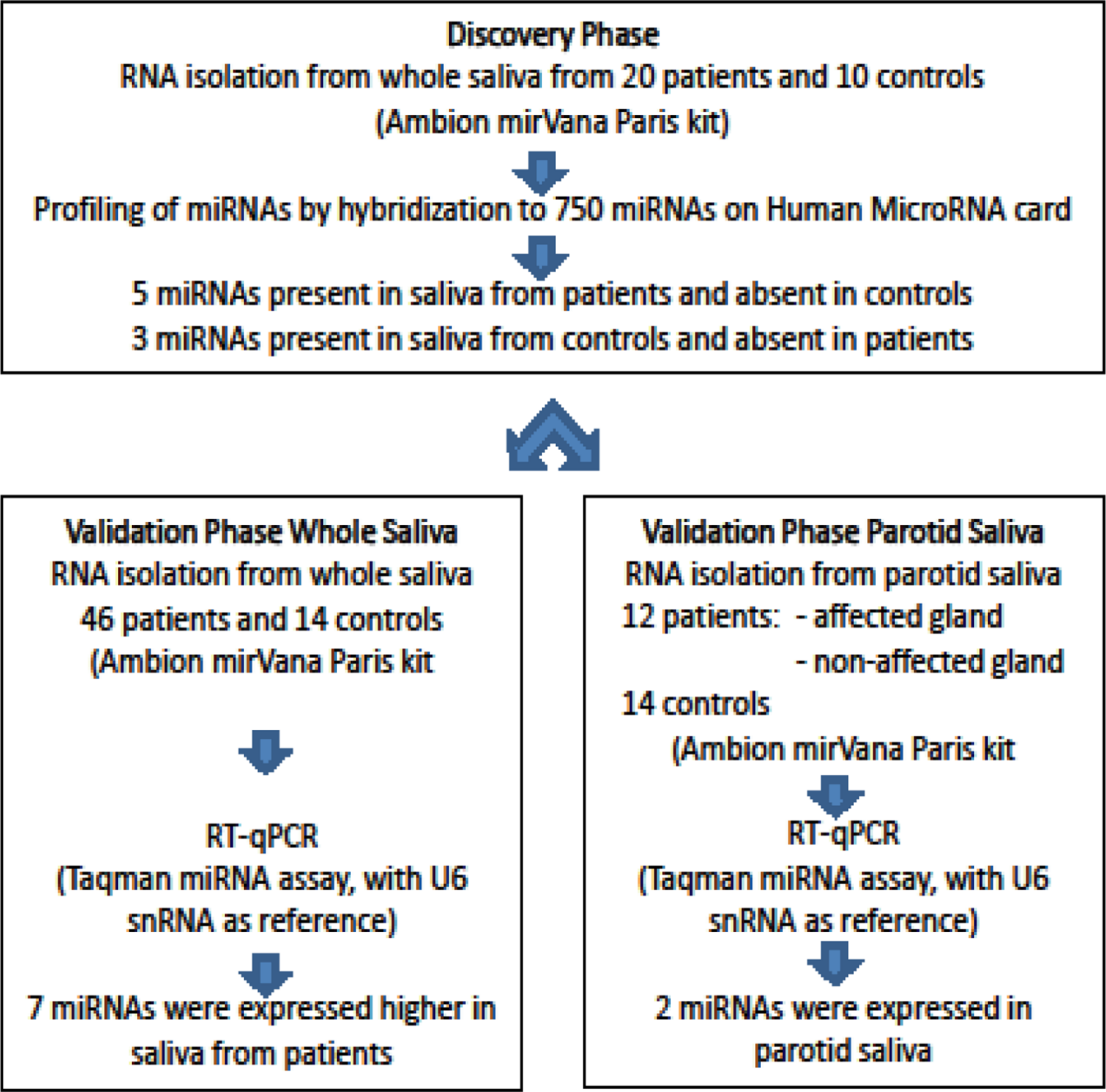
Overview of the research design and saliva samples used in the discovery and validation phase.

The Ct value is defined as the cycle number in the fluorescence emission which exceeds that of a fixed threshold. A Ct of 15–30 was considered high expression, and a Ct of 35 was considered low expression. A Ct value above 40 was considered as undetectable miRNA. We calculated ∆Ct by subtracting the Ct value of the reference gene, RNA polymerase III transcribed U6 snRNA [18], from the Ct value of each candidate biomarker. Data normalization was performed using RQ manager 1.2.1 and Data Assist v3.0 from Applied Biosystems. The qPCR based gene expression values between the two groups were compared using the non-parametric Wilcoxon test. Potential miRNA genes were then selected based on P < 0.05. Fig 1 gives an overview of research design and saliva samples used in the discovery and validation phase.

#### Validation phase in whole saliva

Eight miRNAs which showed the highest difference in expression between the samples from patients with a parotid tumor and normal controls and were overlapping between malignant and benign neoplasms were selected for further testing in the validation phase. The selected miRNAs were validated by RT-qPCR using Taqman miRNA assays in an independent sample set consisting of 46 saliva samples from patients with a parotid salivary gland tumor and 14 saliva samples from controls. RT-qPCR was performed as previously described [16]. Briefly, total RNA was reverse-transcribed and pre-amplified. RT-qPCR was done using specific probes for selected miRNAs and was carried out in a 384 well plate using the Roche LightCycler 480 II (Roche, San Francisco, CA). All RT-qPCRs were performed in duplicate for all candidate miRNAs. U6 snRNA was used as a reference miRNA.

#### Validation of discovered miRNA in parotid saliva

RT-qPCR was used to analyze parotid saliva for the presence of the validated miRNAs. Total RNA was isolated from parotid saliva as described above. Parotid saliva samples were analyzed for the presence of miRNAs using Taqman microRNA assays as described above for validation. All qPCRs were performed in duplicate for all candidate miRNA and negative controls (in which RNA was omitted). For miRNA qPCR experiments, U6 snRNA was used as the reference miRNA.

### Data analysis

In the discovery phase, miRNA profiling data was analyzed for miRNAs that were absent and/or present in whole saliva from patients with a parotid gland neoplasm in comparison to controls. Wilcoxon rank-sum test was performed to compare the expression of miRNAs in samples from patients with a parotid gland neoplasm to controls in order to determine the level of significance for the miRNA biomarkers. The miRNAs emerging from the discovery phase were validated in an independent set of 60 saliva samples from 46 patients with a parotid gland neoplasm and 14 controls. Wilcoxon rank-sum test was used to compare the ∆Ct values for miRNAs between the groups. A two-sided p-value less than 0.05 was deemed statistically significant. Next, multivariate logistic regression analysis was used to construct a classification model to discriminate between patients with a tumor and healthy controls. Backward-stepwise model selection criterion was used to obtain a final model. Specifically, we started fitting a prediction model with all the miRNA biomarkers that differentially expressed between the two groups. In each model reduction step, the miRNA that was the least significant was removed. We continued by successively re-fitting reduced models and applying the same rule until all remaining miRNAs were statistically significant. ROC curves were constructed to determine the diagnostic/predictive values of individual and combined biomarkers from the logistic model. Area under the curve was computed via numerical integration of the ROC curves. The performance of the model for classification was assessed by identifying the cut-off point of the predicted probability which yielded the largest sum of sensitivity and specificity. Leave-one-out cross-validation was used to validate the logistic regression model and the cross-validation error rate was reported. All statistical analyses were performed by statistical software R Version 2.15.0 (http://www.r-project.org/).

### Ethics statement

Permission for this study was obtained from the Medical Ethical Committee of the VU University medical center (registration number: 2009/58), for patients and healthy controls from VU university medical center and the Academic Centre for Dentistry Amsterdam. Also, permission was obtained from the SGTB at MD Anderson Cancer Clinic (IRB: LAB07-0383), the Samsung Medical Center (SMC IRB file #: 2008-01-028-016) and the UCLA Institutional Review Board (UCLA IRB) (ref number: FWA00004642). All participants were adults (> 18 yrs of age) and gave their written informed consent.

## Results

### Discovery of salivary miRNA markers

In the discovery phase, miRNA expression profiles in whole saliva from patients with a parotid gland neoplasm (both benign and malignant) and miRNA expression profiles in whole saliva from controls were established using Taqman Human MicroRNA cards. 742 miRNAs were analyzed (S1 Table). Of these, 5 miRNAs (hsa-miR-296-5p, hsa-miR-577, hsa-miR-1233, hsa-miR-1267, and hsa-miR-1825) had a significantly higher expression level (lower **ΔCt)** in whole saliva samples from patients with a parotid gland neoplasm compared to their expression levels in whole saliva from healthy controls. In addition, 3 miRNAs (hsa-miR-103a-3p, hsa-miR-211 and hsa-miR-425-5p) had a significantly higher expression level in whole saliva from controls compared to patients with a parotid gland neoplasm (Table 2).

**Table 2:** Mean ΔCt (sd) and Wilcoxon 2-sided p-values of discovered salivary miRNA biomarkers. The discovery sample set consisted of whole saliva samples from patients with a parotid gland neoplasm (n = 20) and whole saliva samples from healthy controls (n = 10).

miRNA	ΔCt control mean (sd)	ΔCt neoplasm mean (sd)	Wilcoxon 2-sided p-value
**hsa-miR-103a-3p**	6.34 (1.71)	ND	< 0.001
**hsa-miR-211**	9.45 (1.38)	ND	< 0.001
**hsa-miR-425-5p**	11.56 (3.72)	ND	< 0.001
**hsa-miR-296-5p**	ND	7.41 (3.75)	< 0.001
**hsa-miR-577**	ND	5.96 (3.44)	< 0.001
**hsa-miR-1233**	19.14 (4.98)	8.93 (8.49)	0.002
**hsa-miR-1267**	ND	3.87 (2.79)	< 0.001
**hsa-miR-1825**	20.94 (2.26)	5.17 (5.38)	< 0.001

ND: non-detectable, assigned to samples with a Ct-value of 40 and higher.

### Validation of salivary miRNA markers

**Fig 2:**
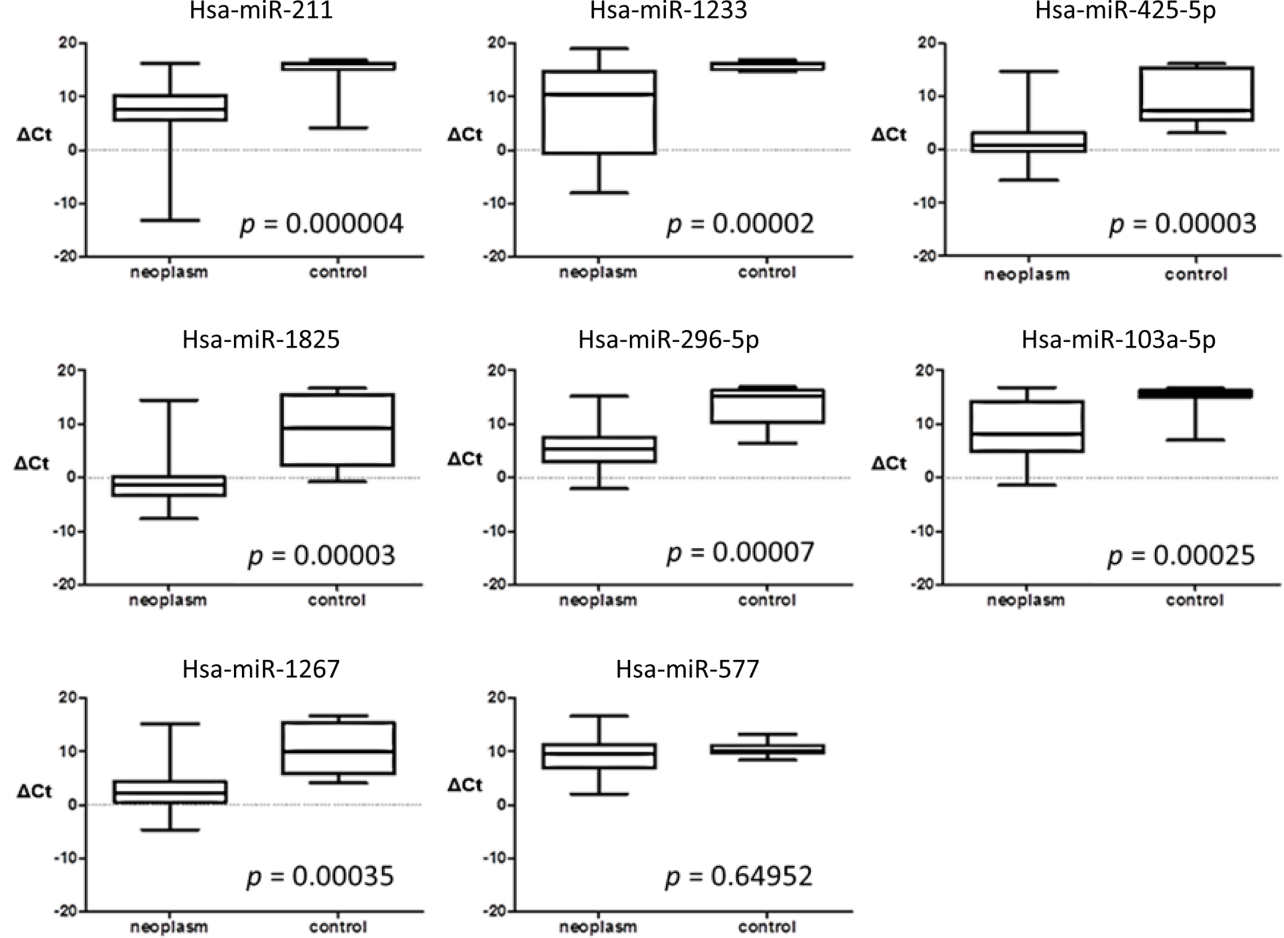
Box-and-whisker plot of the validation of miRNA expression in whole saliva samples. Samples were collected from patients with a parotid gland neoplasm (n = 46) and controls (n = 14). Whiskers represent maximum and minimum ΔCt values. * p < 0. 001.

The expression levels of the 8 miRNAs identified in the discovery phase were validated by RT-qPCR in a separate independent sample set consisting of whole saliva samples from 60 individuals: 46 patients with a parotid gland neoplasm and 14 healthy controls (S2 Table). Four of the five tumor-specific miRNAs (hsa-miR-296-5p, hsa-miR-1233, hsa-miR-1267, and hsa-miR-1825) had a significantly higher expression level in whole saliva from patients than in whole saliva from healthy controls (Table 3). Hsa-miR-103a-3p, hsa-miR-211 and hsa-miR-425-5p,which in the discovery phase were not expressed in saliva from patients, were in the validation phase expressed in saliva from both controls and patients. The expression levels of hsa-miR-103a-3p, hsa-miR-211 and hsa-miR-425-5p were significantly higher in saliva from patients than that from controls (Fig 2 and Table 3). In both the control and tumor groups, some of the whole saliva samples were positive for the investigated miRNAs while others were negative, resulting in a large variation in miRNA expression levels.

**Table 3:** Mean ΔCt (sd) and Wilcoxon 2-sided p-values of validated salivary miRNA biomarkers. The independent validation sample set consisted of whole saliva samples from patients with a parotid gland neoplasm (n = 46) and whole saliva samples from healthy controls (n = 14).

miRNA	ΔCt control mean(sd)	ΔCt neoplasm mean (sd)	Wilcoxon 2-sided p-value
**hsa-miR-103a-3p**	14.32 (3.59)	10.00 (4.75)	< 0.001
**hsa-miR-211**	14.91 (3.18)	8.56 (4.37)	< 0.001
**hsa-miR-296-5p**	12.80 (4.15)	6.95 (4.66)	< 0.001
**hsa-miR-425-5p**	9.96 (5.13)	2.54 (4.79)	< 0.001
**hsa-miR-577**	10.19 (1.57)	9.66 (2.85)	0.650
**hsa-miR-1233**	15.74 (0.70)	9.17 (6.89)	< 0.001
**hsa-miR-1267**	9.78 (5.03)	4.39 (5.21)	< 0.001
**hsa-miR-1825**	7.47 (7.46)	-0.77 (3.95)	< 0.001

### Evaluation of validated miRNA biomarkers

**Fig 3:**
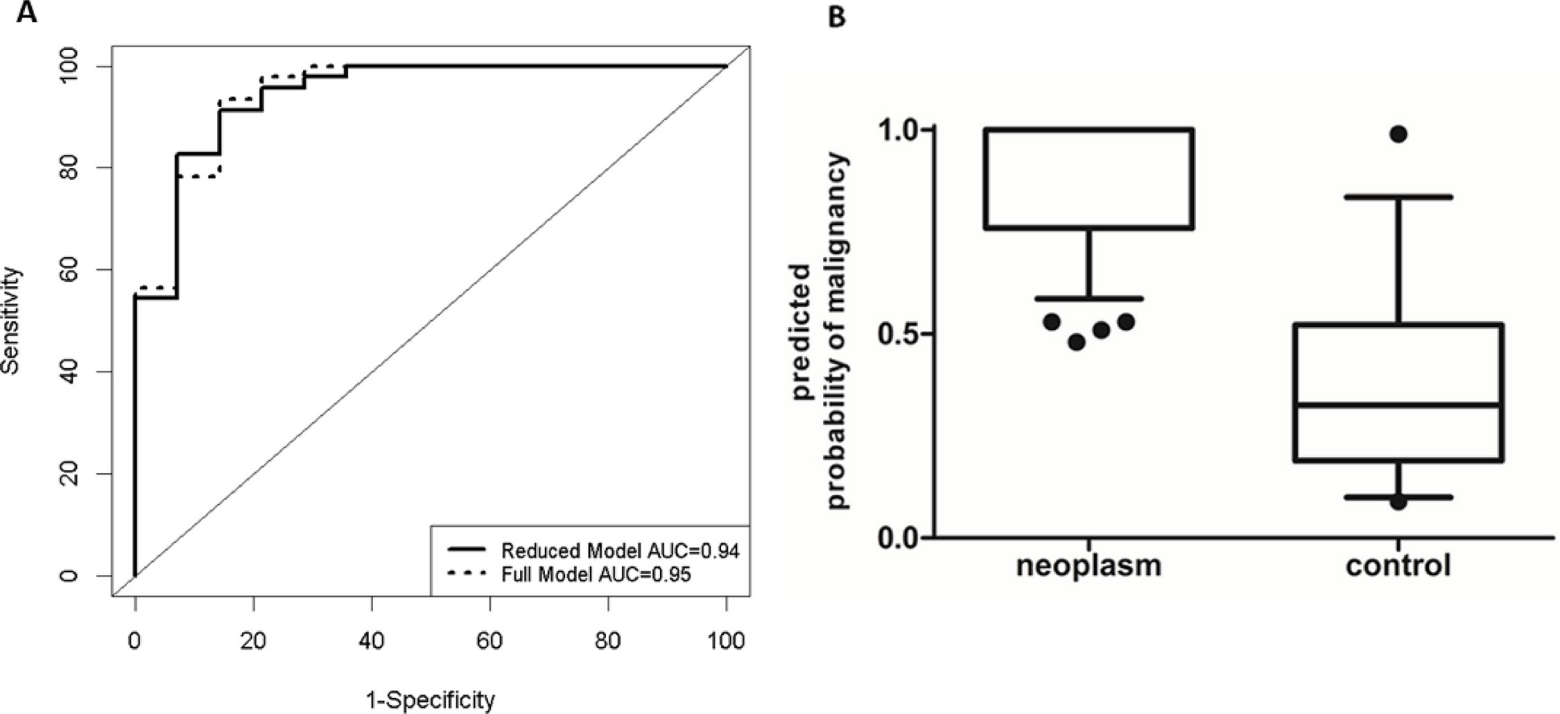
Receiver-operating-characteristics (ROC) curve and dot-plot showing the “predicted probability of neoplasm”. **A.** ROC curves computed from the full (dotted line) and the reduced (solid line) logistic regression models. The full model included 7 miRNAs (hsa-miR-103a-3p, hsa-miR-211, hsa-miR-296-5p, hsa-miR-425-5p, hsa-miR-1233, hsa-miR-1267 and hsa-miR-1825). It yielded an AUC of 0.95 (95% CI: 0.88–1.00), a sensitivity of 93% and a specificity of 86%. The reduced final model included hsa-miR-2 and hsa-miR-1233, which in combination provided satisfactory prediction while being parsimonious. The AUC was 0.94 (95% CI: 0.87–1.00), the specificity was 86% and sensitivity was 91%. **B.** Predicted probability of neoplasm was based on validation data of 2 validated miRNAs (hsa-miR-211 and hsa-miR-1233). Whole saliva samples from patients with a parotid gland neoplasm had a high “predicted probability of neoplasm”, while whole saliva samples from controls with non-neoplastic parotid glands in average had a lower “predicted probability of neoplasm”. Lower line represents minimum “predicted probability of neoplasm”, middle line represents the mean “predicted probability of neoplasm”, and the upper line represents the maximum “predicted probability of neoplasm”.

Multivariate logistic regression models with these miRNA biomarkers were constructed for the classification of patient samples into parotid gland neoplasm and healthy categories. The initial full model consisted of the seven significant miRNAs from Table 3. We then used backward elimination to identify the most parsimonious model. Receiver operating characteristic (ROC) curves were constructed for various possible cut-points of the predicted probability to obtain another measure of the predictive ability of the fitted models. Areas under the curve (AUC) and their 95% confidence interval were computed. The performance of the models were assessed by identifying the cut-off point of the predicted probability which yielded the largest sum of sensitivity and specificity. The full model with 7 miRNA markers (hsa-miR-103a-3p, hsa-miR-211, hsa-miR-296-5p, hsa-miR-425-5p, hsa-miR-1233, hsa-miR-1267 and hsa-miR-1825) yielded an AUC of 0.95 (95% CI: 0.88–1.00), a sensitivity of 93% and a specificity of 86%. On the other hand, the reduced model with 2 miRNAs (hsa-miR-211 and hsa-miR-1233) yielded an AUC of 0.94 (95% CI: 0.87–1.00) a sensitivity of 91% and a specificity of 86%, which provided satisfactory prediction as compared to the full model (Fig 3A). We also performed leave-one-out cross validation on the reduced model. The error rate of leave-one-out cross validation was 0.18.

A box-and-whisker plot predicting the probability of neoplasm (Fig 3B) was constructed based on the validation data of a 2 miRNA combination (hsa-miR-1233 and hsa-miR-211). Most samples of the parotid gland neoplasm group scored a probability of neoplasm of 0.8–1.0. In contrast, the predicting probability of neoplasm of the control samples were spread out, ranging from a probability of neoplasm of 0.09 to 0.99 (Fig 3B).

### The expression of validated miRNAs in parotid saliva

Next, we investigated whether the 7 validated neoplasm-associated miRNAs in whole saliva from both patients and controls were derived from the affected parotid gland. From 12 patients, saliva from both the affected as well as the healthy parotid gland were collected separately. Similarly, saliva from both parotid glands was collected from 14 healthy controls. Whole saliva samples from these patients and controls had already been analyzed in the validation phase. Two of the 7 validated miRNAs (hsa-miR-425-5p and hsa-miR-1825) were expressed in parotid saliva. The expression levels of these miRNAs differed only marginally between parotid saliva of patients and controls, or, within patients, between saliva from the affected and the non-affected parotid glands. However, the expression level of miRNAs in parotid saliva samples from patients was much lower than in their corresponding whole saliva samples (Table 4). The expression level of hsa-miR-425-p and hsa-miR-1825 in parotid saliva from healthy controls was similar to the expression level of hsa-miR-425-5p and hsa-miR-1825 in whole saliva from healthy controls.

**Table 4:** Mean ΔCt (sd) of validated salivary miRNA biomarkers in whole saliva and parotid saliva from patients and controls.

	Mean ΔCt (sd) in saliva from patients (n = 12)			Mean ΔCt (sd) in saliva from controls (n = 14)		p-value				
miRNA	Affected parotid saliva	non-affected parotid saliva	whole saliva	whole saliva	parotid saliva	affected parotid vs non-affected parotid saliva	affected parotid vs control parotid saliva	non-affected parotid vs control parotid saliva	affected parotid vs control whole saliva	patients whole vs control whole saliva
**hsa-miR-103a-3p**	15.25 (0.72)	14.46 (0.99)	13.33 (2.87)	14.92 (2.79)	14.32 (3.59)	0.04*	0.63	0.18	0.38	0.03*
**hsa-miR-211**	15.09 (0.53)	15.39 (0.95)	10.31 (4.18)	14.48 (2.90)	14.91 (3.18)	0.31	0.7	0.37	0.06	<0.001*
**hsa-miR-296-5p**	14.04 (2.71)	13.96 (3.21)	9.43 (4.92)	13.97 (2.63)	12.80 (4.15)	0.67	0.98	0.63	0.98	0.02*
**hsa-miR-425-5p**	8.29 (4.44)	9.61 (3.83)	4.23 (4.21)	10.56 (5.43)	9.96 (5.12)	0.34	0.18	0.59	0.32	0.01*
**hsa-miR-1233**	15.24 (0.72)	15.39 (0.95)	11.54 (7.09)	15.30 (0.76)	15.74 (0.70)	0.54	0.98	0.37	0.09	0.01*
**hsa-miR-1267**	14.18 (0.97)	14.33 (1.07)	7.24 (4.27)	13.61 (5.03)	9.78 (5.03)	0.62	0.28	0.49	0.16	0.2
**hsa-miR-1825**	5.87 (3.79)	6.51 (3.45)	0.06 (1.51)	6.26 (1.51)	7.47 (4.66)	0.62	0.55	0.29	0.4	<0.001*

## Discussion

In this study, we investigated and validated differences of miRNA expression levels in saliva from patients with a parotid gland neoplasm and healthy controls. The expression of miRNA in whole saliva from patients with a parotid gland neoplasm differs from the expression of miRNA in whole saliva from controls. The expression level of 7 miRNAs was higher in saliva from patients compared to controls and could be confirmed in an independent sample set. A combination of two of these miRNAs (hsa-miR-1233 and hsa-miR-211) enabled a predictive value with a sensitivity of 91%, and a specificity of 86% for predicting the presence of a parotid gland neoplasm.

Today, the invasive fine-needle aspiration cytology (FNAC) technique is one of the most commonly used techniques for the diagnosis of a swelling of the salivary gland. A systematic review with regard to the performance of FNAC in parotid gland lesions concluded that FNAC had a specificity of 97% and a sensitivity of 80%. However the performance variability was relatively large [19]. Therefore, additional (molecular) markers, such as those identified in the present study, can add to the accuracy of a pre-operative diagnosis by FNAC. One of these molecular markers can be salivary miRNA. MiRNAs are stably expressed in all body fluids [2], and are also less prone to being degraded, unlike mRNA or proteins. The ease of saliva sampling, the stable expression of miRNAs and the fact that miRNAs are less prone to be degraded make salivary miRNAs a good choice for biomarkers [20,21].

Strikingly, we observed differences in the expression of miRNA between the discovery and validation phases. In the discovery phase, hsa-miR-103a-3p, hsa-miR- 211 and hsa-miR-425-5p were expressed higher in whole saliva samples from controls compared to whole saliva samples from patients. In contrast, these miRNAs had a higher expression level in whole saliva from patients compared to controls in the validation phase. Such differences in miRNA expression have been shown before, highlighting the importance and necessity of validating biomarkers in an independent sample set [22]. Inconsistency between findings of different study groups may be due to a large variation in expression levels of miRNA in controls and patients, or possibly due to variation in miRNA expressions between different ethnic groups as our samples were derived from various sources in both phases of the study. Moreover, we used different histological types of parotid neoplasms, both benign and malignant, which can also be a source of variation, although the relative frequency of the neoplasms was comparable between the discovery and validation phase.

Because miRNAs are known to have a gene regulatory role, we conducted an unbiased search (www.miRBase.org) as to whether the discovered miRNAs had validated targets. Three of the discovered miRNAs (hsa-miR-211, hsa-miR-296-5p, and hsa-miR-425-5p) had validated targets. It has been shown that hsa-miR-211 targets tumor suppressor cadherin 5, which promotes colorectal cancer cell growth in vitro and in vivo [23]. In oral carcinomas, the elevated expression of hsa-miR-211 is associated with poor prognosis [24]. One of the validated targets for hsa-miR-296-5p is the B-cell lymphoma 2 gene, (*BCL2*) a member of the *Bcl2*-family of apoptosis regulator proteins. A high expression of hsa-miR-296-5p may result in suppression of *BCL2* transcription which, in turn, could lead to higher rates of cell death. One of the many targets of hsa-miR-425-5p is Dicer1, a RNase endonuclease III enzyme that is necessary for miRNA maturation. A study describing the expression of Dicer1 in mucoepidermoid carcinoma found that Dicer1 was expressed higher in MEC compared to surrounding normal tissue [25]. The targets for hsa-miR-211, hsa-miR-296-5p and hsa-miR-425-5p have only been validated in tissue and not in saliva itself. Therefore, we can only speculate what role hsa-miR-211, hsa-miR-296-5p and hsa-miR-425-5p play in saliva.

The present study revealed that only 2 of the 7 validated miRNAs (hsa-miR-425-5p and hsa-miR-1825) were expressed in parotid saliva from patients and controls. The expression of hsa-miR-103a-3p, hsa-miR-211, hsa-miR-296-5p, hsa-miR-1233 and hsa-miR-1267 in whole saliva and the absence of these miRNAs in parotid saliva suggest that these miRNAs are not specifically expressed and/or secreted from the parotid gland tumor. The differences in miRNA expression which we observed between unstimulated whole saliva (Table 3) and stimulated parotid saliva (Table 4) suggest that parotid saliva may have a totally different miRNA expression profile. Other studies have shown that tumors express miRNAs that may be involved in intercellular crosstalk [26,27]. This fits with the hypothesis in which tumor cells transport genetic material, including miRNA, via micro-vesicles (exosomes) to neighboring and/or distant cells, thereby affecting the miRNA expression of those cells and supporting cell growth and progression [9,28]. The miRNAs which are present in blood may leak from the gingival crevice into the oral cavity. This provides whole saliva with many, if not most, of the same molecules found in the systemic circulation including miRNAs.

The initial data presented in this study are a first step toward developing a clinical application to distinguish parotid salivary gland tumors from healthy parotid glands. Future studies should be directed to further unravel the differences between whole saliva and parotid saliva. Although whole saliva is more easily accessible, we cannot conclude, at this moment, which source gives more robust data for tumor diagnostics. Moreover, in the future, differences in miRNA expression in different histological tumor types should also be taken into consideration. Our results justify and urge further investigation towards the discovery of potentially relevant miRNAs in saliva.

## Supporting Information

S1 Table Average Ct and average ΔCt values from the discovery phase. In the discovery phase, the expression of miRNAs in whole saliva from patients with a parotid gland neoplasm (n = 20) were compared to the expression of miRNAs in whole saliva from controls (n = 10).
(XLSX)

S2 Table Average Ct and average ΔCt values from the validation phase. Data of the validation of 8 miRNAs in an independent sample set consisting of 46 whole saliva samples from patients with a parotid gland neoplasm and 14 whole saliva samples from controls.
(XLSX)
