# Correction: Human Salivary Micro-RNA in Patients with Parotid Salivary Gland Neoplasms

**DOI:** 10.1371/journal.pone.0146701

**Published:** 2016-01-05

**Authors:** Johannes H. Matse, Janice Yoshizawa, Xiaoyan Wang, David Elashoff, Jan G. M. Bolscher, Enno C. I. Veerman, C. René Leemans, D. Michiel Pegtel, David T. W. Wong, Elisabeth Bloemena

The eighth author’s name is spelled incorrectly. The correct name is: D. Michiel Pegtel.
